# A case report of confirmed difficult pulmonary tuberculosis based on the hybrid capture-based tNGS method

**DOI:** 10.1186/s12890-025-03539-7

**Published:** 2025-02-06

**Authors:** Weiqian Chen, Huimin Chen, Ze Liu, Xinle Chi, Yaomeng Chen, Huan Ye, Wenjie Huang, Chenlei Cao, Wei Weng

**Affiliations:** 1 Department of Respiratory Medicine, The Wenzhou Third Clinical Institute Affiliated to Wenzhou Medical University, Wenzhou People’s Hospital, Wenzhou 325000 Zhejiang, China; 2 Department of Cardiology, Wenzhou TCM Hospital of Zhejiang Chinese Medical University, Wenzhou 325000 Zhejiang, China; 3 Department of Nuclear Medicine, Ningbo Hangzhou Bay Hospital, Ningbo, 315327 Zhejiang China; 4 Department of Radiology, The Wenzhou Third Clinical Institute Affiliated to Wenzhou Medical University, Wenzhou People’s Hospital, Wenzhou 325000 Zhejiang, China; 5 Department of Nuclear Medicine, Xinqiao Hospital, Chongqing, 400037 China

**Keywords:** Pulmonary tuberculosis, Hybrid capture-based tNGS, Diagnostic methods, Case report

## Abstract

**Background:**

Early diagnosis of pulmonary tuberculosis can greatly reduce the harm caused by the disease. However, traditional diagnostic methods have various shortcomings in diagnosing pulmonary tuberculosis. Currently, with the increasing popularity, iteration, and decreasing costs of Next-generation sequencing (NGS) testing technology, NGS is being more widely applied in the diagnosis of pulmonary tuberculosis.

**Case presentation:**

A 29-year-old male presented with “fever accompanied by cough for more than 20 days.” Multiple chest CT scans revealed progressive enlargement of the right hilar lymph nodes and thickening of the interlobular septa in the right upper lobe. Routine testing of bronchoalveolar lavage fluid, search for tuberculosis bacilli, bacterial and fungal cultures, X-pert MTB/RIF, and multiplex PCR-based targeted Next-generation sequencing (mp-tNGS) results were all inconclusive. Finally, bronchoalveolar lavage fluid was sent for hybrid capture-based targeted Next-generation sequencing (hc-tNGS) testing, and special staining of the enlarged lymph nodes confirmed the diagnosis of pulmonary tuberculosis.

**Conclusion:**

The hc-tNGS has significant value in diagnosing pulmonary tuberculosis, especially in cases that are difficult to detect with other methods. In the future, this could gradually become a routine diagnostic method for pulmonary tuberculosis, enhancing the accuracy of early diagnosis.

**Supplementary Information:**

The online version contains supplementary material available at 10.1186/s12890-025-03539-7.

## Background

**Fig. 1:**
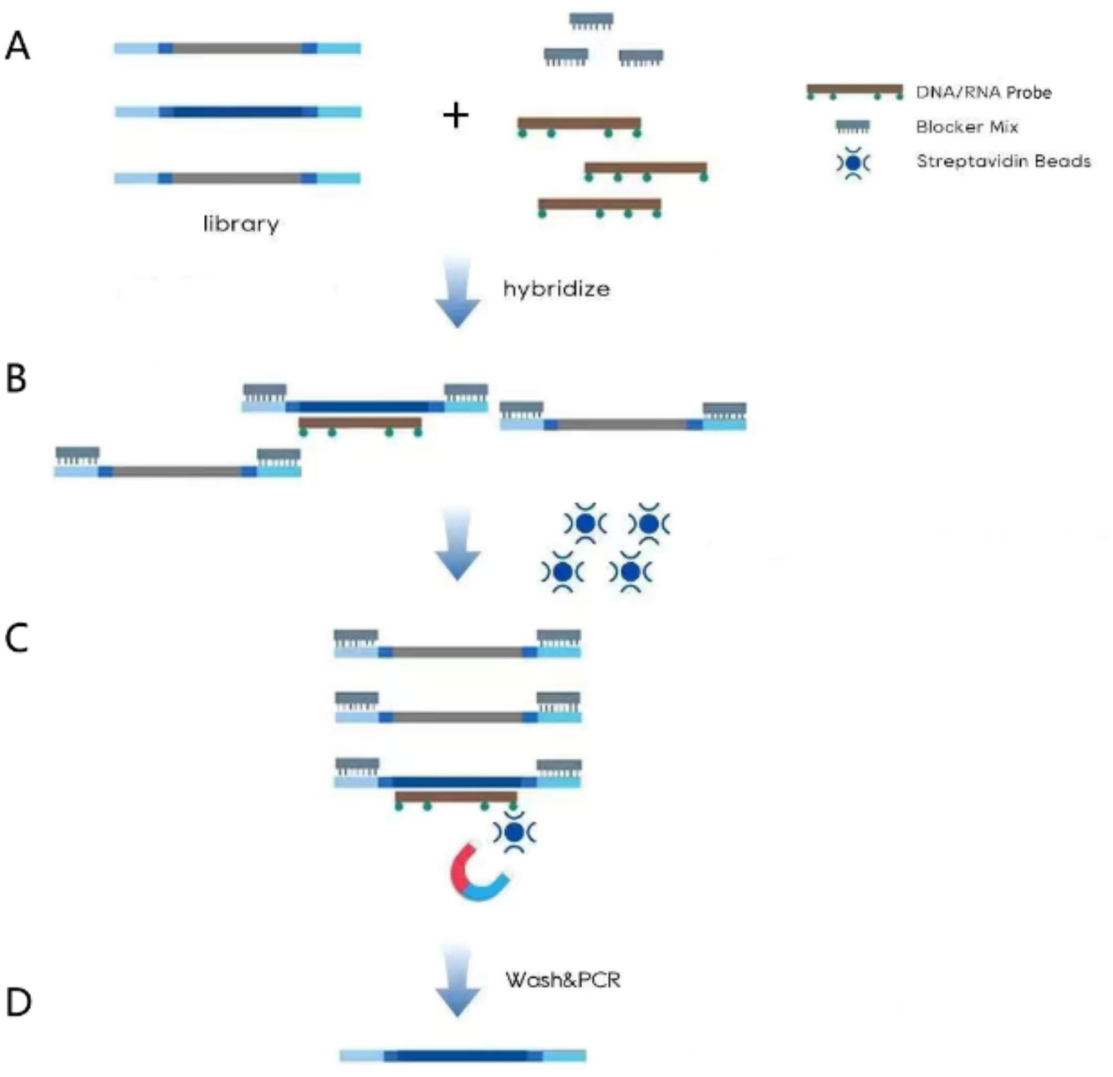
(**A**) The adapter ligation buffer is used to seal the two ends of the nucleic acid library to prevent the formation of primer dimers. (**B**) The capture probe (with biotin) binds to target pathogens with species-specific sequences. (**C**) Magnetic beads coated with streptavidin are added to bind with the biotin-labeled capture probes, which are then extracted using a magnet to isolate the magnetic bead-capture probe-target sequence complex. (**D**) Heating is applied for elution to obtain the target sequence

Tuberculosis remains one of the most serious public health issues globally. The World Health Organization (WHO) reported in 2023 that an estimated 10.6 million cases of tuberculosis occurred worldwide in 2022, indicating that the epidemic is still severe [1]. The lungs are the most common site of infection by Mycobacterium tuberculosis, and early diagnosis and treatment are crucial for controlling the disease. Traditional diagnostic methods for pulmonary tuberculosis include sputum smear for tuberculosis bacilli and culture of the tuberculosis bacteria [2–4]. However, these traditional methods have various shortcomings, such as prolonged diagnostic times and inadequate sensitivity and specificity. In recent years, advancements in diagnostic technologies, including T-SPOT, X-pert MTB/RIF, Tuberculosis loop-mediated isothermal amplification (TB-LAMP), and NGS, have significantly improved the diagnostic capabilities for pulmonary tuberculosis, with NGS demonstrating the most remarkable efficiency. The NGS technology continues to evolve, moving from the initial metagenomic Next-generation sequencing (mNGS) and multiplex PCR-based targeted Next-generation sequencing (mp-tNGS) to a hybrid capture-based targeted Next-generation sequencing (hc-tNGS) that incorporates probes. The hc-tNGS is relatively rare in clinical applications. This method utilizes a panel of oligonucleotide probes to capture nucleotide sequences of interest, followed by next-generation sequencing (NGS) of the enriched targets for sensitive detection and sequence-based analysis [5]. Its specific mechanism of action is illustrated in Fig. 1. In this case of difficult-to-diagnose pulmonary tuberculosis, various diagnostic methods failed to confirm the diagnosis until the hc-tNGS test was conducted, which confirmed the presence of tuberculosis. This indicates that hc-tNGS has great potential for diagnosing pulmonary tuberculosis.

## Case presentation

**Fig. 2:**
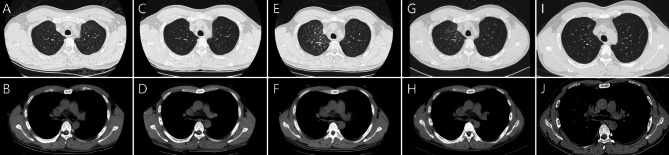
(**A**,** B**) Chest CT on April 12, 2024, shows enlarged right hilar lymph nodes, measuring approximately 21.63 mm × 21.14 mm. (**C**,** D**) Chest CT on April 20, 2024, shows enlarged right hilar lymph nodes, measuring approximately 22.80 mm × 22.75 mm. (**E**,** F**) Chest CT on April 25, 2024, shows enlarged right hilar lymph nodes, measuring approximately 31.66 mm × 23.85 mm, with thickening of the interlobular septa in the right upper lung. (**G**,** H**) Chest CT on April 29, 2024, shows enlarged right hilar lymph nodes, measuring approximately 32.75 mm × 21.64 mm, with thickening of the interlobular septa in the right upper lung. (**I**,** J**) Chest CT on September 25, 2024, showed significant absorption of the pulmonary lesions after antituberculosis treatment, and the hilar lymph nodes had decreased in size, measuring approximately 25.19 mm × 15.68 mm

**Fig. 3:**
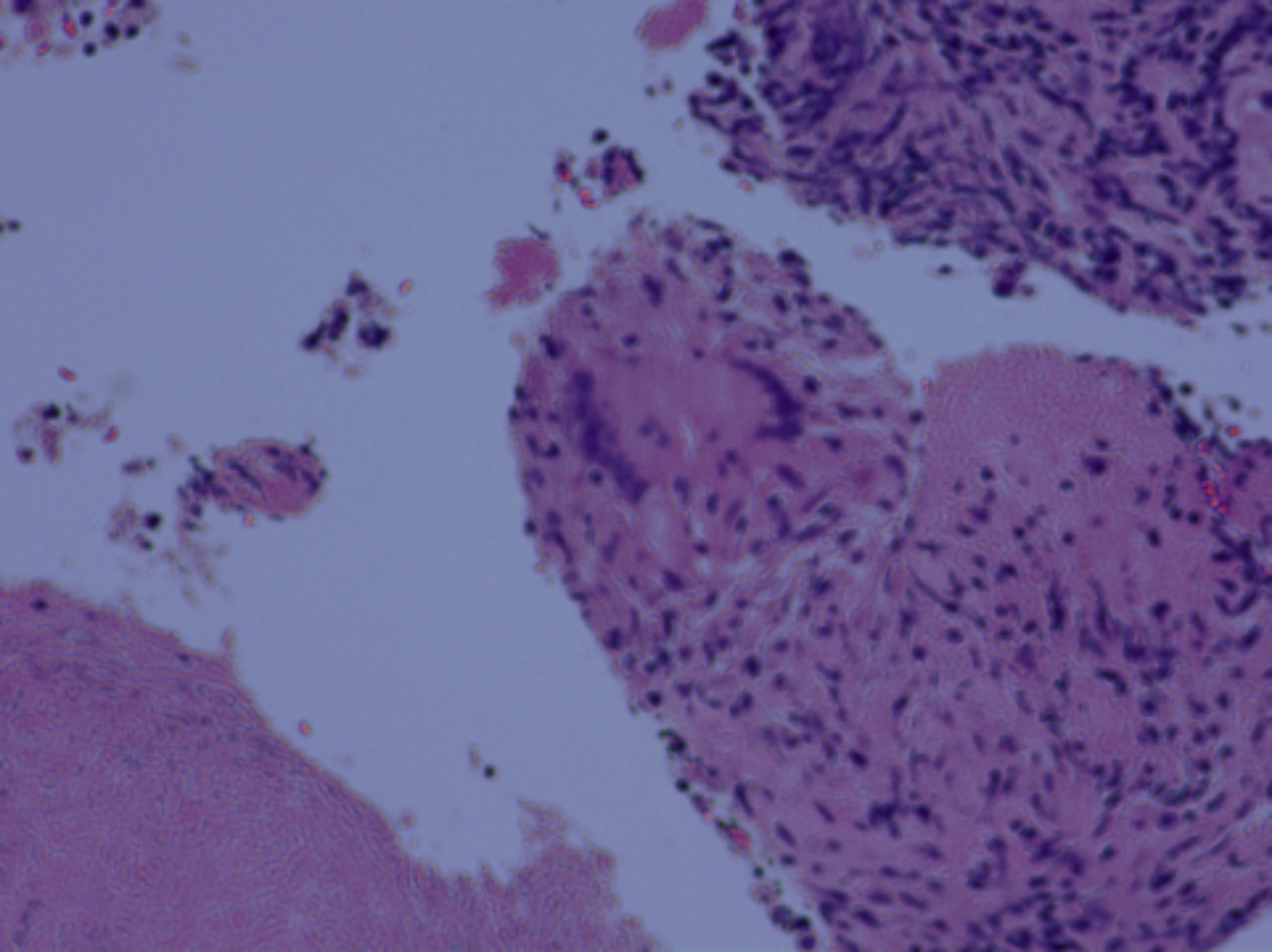
Pathological result: necrotizing granuloma

A 29-year-old male was admitted due to “fever accompanied by cough for more than 20 days.” The patient developed a fever and cough over 20 days ago without any obvious cause, with a temperature of approximately 37.8℃. He had a history of good health and denied having hypertension, diabetes, infectious diseases, as well as any history of surgery or trauma. He sought treatment at another hospital, where he received anti-infective and symptomatic treatment; however, his clinical symptoms showed no significant improvement. During his treatment period (from April 10 to April 25, 2024), chest CT scans indicated progressive enlargement of the right hilar lymph nodes and newly developed thickening of the interlobular septa in the right upper lobe (Fig. 2A and F). Upon arrival at our hospital, the physical examination showed smooth breathing, clear lung sounds bilaterally, no obvious dry or wet rales, and no significant abnormalities. Laboratory tests revealed a white blood cell count of 9.0 × 10^9^/L, a C-reactive protein level of 41.9 mg/L↑, an erythrocyte sedimentation rate of 35 mm/h↑, negative tumor markers, and a T-SPOT result of > 12.00 pg/ml↑. The chest contrast enhanced CT scan shows that the lymph node has slightly enlarged compared to April 25, 2024 (Fig. 2G and H). We performed bronchoscopy, conducting routine tests on the bronchoalveolar lavage fluid, searching for Mycobacterium tuberculosis, bacterial and fungal cultures, X-pert MTB/RIF, and mp-tNGS examinations; however, all results were inconclusive. Subsequently, Endobronchial ultrasound-guided transbronchial needle aspiration (EBUS-TBNA) was performed to obtain lymph node tissue, and the pathology results indicated necrotizing granuloma (Fig. 3). This series of results excluded the possibility of pulmonary tuberculosis, which was inconsistent with clinical expectations. Ultimately, the patient’s bronchoalveolar lavage fluid underwent hc-tNGS testing, and the lymph node pathology was subjected to acid-fast staining. The hc-tNGS results indicated a positive presence of Mycobacterium tuberculosis, and subsequent special staining of the lymph node pathology confirmed the diagnosis of tuberculosis infection. The patient underwent a follow-up chest CT after 5 months of regular treatment for tuberculosis. The chest CT showed significant absorption of the pulmonary lesions, and the hilar lymphadenopathy had also reduced (Fig. 2I and J).

## Discussion

The lungs are the most common site for infection by Mycobacterium tuberculosis, with nearly 75% of tuberculosis patients affected in this area [3]. The clinical symptoms of pulmonary tuberculosis are non-specific and can manifest as cough, sputum production, hemoptysis, afternoon low-grade fever, night sweats, and weight loss [6]. Pulmonary tuberculosis can be classified into primary, hematogenous disseminated, and secondary types, with CT findings varying by type [6, 7]. In this case, the CT appearance is highly atypical, showing multiple enlarged lymph nodes in the right hilum and mediastinum, as well as thickening of the interlobular septa in the right upper lobe accompanied by small nodules distributed within the lymphatic interstitium. These imaging findings are not commonly seen and can be observed in various diseases, including primary lung tuberculosis, sarcoidosis, pneumoconiosis, and pulmonary malignancies. Upon the patient’s admission, we considered the potential diagnosis of primary pulmonary tuberculosis. However, primary pulmonary tuberculosis is more commonly seen in children and typically presents with pulmonary lesions first, followed by lymphangitis and enlargement of the hilar lymph nodes. In this patient, lymph node enlargement in the hilum appeared first, followed by thickening of the interlobular septa in the right upper lobe, thus ruling out this diagnosis. We also needed to exclude the possibility of sarcoidosis; although the classic imaging finding for sarcoidosis is symmetrical enlargement of the hilar lymph nodes bilaterally, some patients may present with unilateral hilar lymph node enlargement [8]. Moreover, the results from the X-pert MTB/RIF and mp-tNGS also directed us to consider non-infectious lesions. Without the hc-tNGS and subsequent special staining results from pathology, we might have mistakenly identified this patient as having sarcoidosis, potentially leading to corticosteroid treatment, which would have further exacerbated the tuberculosis infection.

The laboratory tests for this patient revealed that only the T-SPOT result was positive. T-SPOT is one type of IGRA test, which has a higher sensitivity compared to another type, QFT-Plus. It has been previously thought that T-SPOT cannot distinguish between latent tuberculosis infection (LTBI) and active tuberculosis, nor can it be used to diagnose active tuberculosis. A positive T-SPOT result does not necessarily indicate the presence of active tuberculosis, and a negative result does not rule it out either [9, 10]. For example, we have encountered many patients in clinical practice with positive T-SPOT result who ultimately proved not to have LTBI or active tuberculosis [11, 12]. However, this case reminds us that for patients with a positive T-SPOT result, the possibility of active tuberculosis should still be considered, even in the absence of other evidence.

The hc-tNGS has higher enrichment efficiency, covering more pathogens than mp-tNGS and approaching mNGS levels. It also encompasses pathogens that are not enriched and has high specificity, making it suitable for testing common clinical samples [13–18].The diagnosis and treatment process of this patient seems to confirm that its sensitivity is higher than that of mp-tNGS. Of course, this requires further validation through large-scale data. Hc-tNGS also has some of the drawbacks. Firstly, its cost remains relatively high compared to traditional diagnostic methods and mp-tNGS. Secondly, Like other NGS results, interpreting hc-tNGS results poses a challenge, especially in distinguishing whether the detected pathogens are pathogenic, colonizers, or contaminants.

With the widespread adoption and improvement of molecular testing technologies like NGS, the accurate diagnosis of infectious diseases has become increasingly simple and efficient. In diagnosing pulmonary tuberculosis, the hc-tNGS demonstrates higher sensitivity and specificity compared to other testing methods [19, 20]. For patients with a high clinical suspicion of tuberculosis but negative results on other indicators, the hc-tNGS would be an excellent diagnostic option. Although pathological special staining is also very accurate in diagnosing tuberculosis, obtaining tissue biopsies is an invasive procedure and results take longer to obtain. Nevertheless, the diagnostic advantages of the hc-tNGS for pulmonary tuberculosis still require further validation.

## Electronic supplementary material

Below is the link to the electronic supplementary material.

Supplementary Material 1

## Data Availability

The datasets generated and analysed during the current study are available in the Sequence Read Archive, https://www.ncbi.nlm.nih.gov/sra/?term=SRR32129069.

## Abbreviations

EBUS-TBNA: Endobronchial ultrasound-guided transbronchial needle aspiration
hc-tNGS: Hybrid capture-based targeted Next-generation sequencing
IGRA: Interferon-gamma release assay
LTBI: Latent tuberculosis infection
mNGS: Metagenomic Next-generation sequencing
mp-tNGS: Multiplex PCR-based targeted Next-generation sequencing
NGS: Next-generation sequencing
QFT-Plus: QuantiFERON-TB Gold Plus
TB-LAMP: Tuberculosis loop-mediated isothermal amplification
tNGS: Targeted Next-generation sequencing
